# Segmentation of Intensity Inhomogeneous Brain MR Images Using Active Contours

**DOI:** 10.1155/2014/194614

**Published:** 2014-07-16

**Authors:** Farhan Akram, Jeong Heon Kim, Han Ul Lim, Kwang Nam Choi

**Affiliations:** ^1^Department of Computer Engineering and Mathematics, Rovira i Virgili University, 43007 Tarragona, Spain; ^2^Korea Institute of Science & Technology Information, Daejeon 305-806, Republic of Korea; ^3^Department of Computer Science & Engineering, Chung-Ang University, Seoul 156-756, Republic of Korea

## Abstract

Segmentation of intensity inhomogeneous regions is a well-known problem in image analysis applications. This paper presents a region-based active contour method for image segmentation, which properly works in the context of intensity inhomogeneity problem. The proposed region-based active contour method embeds both region and gradient information unlike traditional methods. It contains mainly two terms, area and length, in which the area term practices a new region-based signed pressure force (SPF) function, which utilizes mean values from a certain neighborhood using the local binary fitted (LBF) energy model. In turn, the length term uses gradient information. The novelty of our method is to locally compute new SPF function, which uses local mean values and is able to detect boundaries of the homogenous regions. Finally, a truncated Gaussian kernel is used to regularize the level set function, which not only regularizes it but also removes the need of computationally expensive reinitialization. The proposed method targets the segmentation problem of intensity inhomogeneous images and reduces the time complexity among locally computed active contour methods. The experimental results show that the proposed method yields better segmentation result as well as less time complexity compared with the state-of-the-art active contour methods.

## 1. Introduction

Image segmentation is a fundamental problem in the areas of computer vision and image processing. It is used to partition an image into two or more than two nonoverlapping regions based on textual, intensity, or gradient information. Image segmentation is a particularly difficult task for numerous reasons. Firstly, partitioning the image into nonoverlapping regions and extracting regions of interest requires a tradeoff between the simplicity of algorithm, selection of parameters, computational efficiency of algorithm, and accuracy of the segmentation result. Secondly, image artifacts, such as noise, intensity inhomogeneity, artifacts involved with the image acquisition, and poor contrast of image, are very difficult to account for in segmentation algorithms without high level of interactivity from the user. Different methods are devised in a context of the segmentation problem and each of them has their own advantages and disadvantages. Some of the common techniques used for image segmentation are thresholding based segmentation, segmentation based on image classification, and edge based and region based (region growing) image segmentation.

Active contour is one of the devised techniques for image segmentation problem, which segments an image by evolving a level set curve. In late 1980s, Kass et al. introduced one of the image segmentation techniques based on active contour [1]. In this method, a curve evolves toward the object boundary under a force, until it stops at the boundary. To be more specific, the curve moves toward the object boundaries by minimizing the energy. The energy functional is based on different image characteristics, for example, image gradient, curvature, and image statistical properties.

The existing active contour models are categorized into two groups: edge-based models [1–4] and region-based models [5–14]. Both of these types have their own paybacks and drawbacks, and the choice between them to use in applications depends on the different characteristics of the images.

The edge-based model builds an edge indicator function using image edge information, which can drive the contour towards the object boundaries [2]. The edge indicator function based on the image gradient can hardly stop at the right boundaries, for the images with intense noise or a weak edge.

On the other hand, a region-based model uses statistical information to construct a region stopping function that can stop the contour evolution between different regions. One of the early efforts towards a region-based active contours was made by the Mumford and Shah segmentation model [5], which approximates a given image by a piecewise smooth image. Compared to the edge-based model, the region-based model can perform better for images with blurred edges. The region-based model is not sensitive to initialization of the level set function and can recognize the object's boundaries efficiently. Therefore, region-based models, especially the Chan and Vese (CV) model [6], have been widely applied for image segmentation.

**Figure 1:**
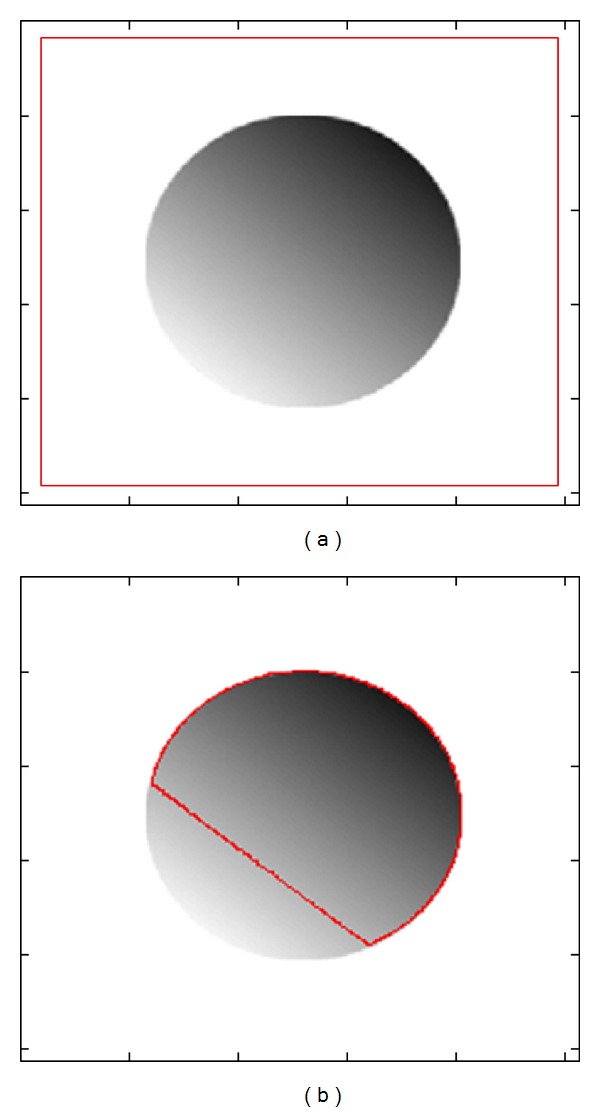
Intensity inhomogeneity problem in segmentation. (a) Image with intensity inhomogeneity; (b) segmentation results using CV energy model.

Although the region-based model is better than edge-based model in some aspects, it still has limitations. The traditional region-based models [5, 6], which were proposed in the context of binary images with the assumption that each image region is statistically homogeneous, do not work perfectly for images with intensity inhomogeneity.  Figure 1(a) shows an image with white background which contains intensity inhomogeneous region in it and Figure 1(b) shows the ineffectiveness of traditional region-based active contour method in case of image with intensity inhomogeneity.

The traditional region-based models [5, 6], which were proposed in the context of binary images, do not work well if the target image contains intensity inhomogeneous regions in it. Li et al. [11, 12] proposed the LBF model by embedding the local image information. LBF model is able to segment images with intensity inhomogeneity and is much more accurate than the previously formulated methods. The basic idea of LBF was to introduce a Gaussian kernel function in the energy functional formulation. Although it segments well the images with intensity inhomogeneity, it has quite high computational time complexity. Therefore, segmentation process takes quite a time as compared to old segmentation methods. Zhang et al. [13] proposed an active contour method driven by local image fitting (LIF) energy, which provides almost same segmentation results and has less time complexity as compared to LBF model.

In this paper, we proposed a region based active contour method which works well under the intensity inhomogeneity problem. The proposed region based active contour model utilizes both edge and region information to segment an image into nonoverlapping region. It is implemented by replacing the edge indicator function in the area term of the edge-based level set method [3] with a new region-based signed pressure force (SPF) function that utilizes the image local information obtained using the local binary fitted (LBF) energy model. By introducing the SPF function based on local fitted image (LFI), the formulated method is able to segment images with intensity inhomogeneity. In the proposed model, region information used in the area term helps to stop the contour at weak or blur edges while edge information in the length term accelerates the detection of those weak or blur edges in corporation of that area term. As the introduced model contains both edge and region information it works better than the traditional edge-based and region-based methods.

Reinitialization, a technique for occasionally reinitializing the level set function to a signed distance function (SDF) during the evolution, has been extensively used as a numerical remedy for maintaining stable curve evolution and ensuring desirable results. From a practical viewpoint, the reinitialization process can be quite convoluted and expensive and has subtle side effects [15]. Zhang et al. [7] proposed the active contour with selective local or global (ACSLG) segmentation method which uses a Gaussian kernel to regularize the level function after each iteration. It not only regularizes the level set but also removes the need of reinitialization. In the proposed algorithm we also use the Gaussian kernel to eliminate the need of reinitialization. Regularization using Gaussian kernel has better smoothing results and no energy leakage as compared to the area smoothing and penalization terms used by Li et al. [3].

The proposed segmentation algorithm is applied to synthetic and brain MR images in order to demonstrate the accuracy, effectiveness, and robustness of the algorithm. A comparison is shown with previous related methods to show advantages of the proposed method.

## 2. Active Contour Method Driven by Locally Computed Signed Pressure Force (LCSPF) Function

Li et al. [11, 12] proposed the LBF energy model by employing the local image information. LBF is able to segment image with intensity inhomogeneity and provides much more accurate results than the traditional region-based methods. The basic idea is to introduce a kernel function to define the LBF energy functional. Let *I* : Ω → *R* be an input image and let *C* be a closed curve, for that the LBF energy functional, *E*_LBF_(*C*, *f*_1_, *f*_2_), is defined as follows:

(1) ELBF(C,f1,f2) =λ1∫ΩKσ1(x−y)|I(y)−f1(x)|2Hε(ϕ(y))dy  +λ2∫ΩKσ1(x−y)|I(y)−f2(x)|2(1−Hε(ϕ(y)))dy.

Minimizing the above energy functional with respect to *f*_1_ and *f*_2_ using steepest gradient descent method [16] we get the following formulations:

(2) f1(x)=Kσ1∗[Hε(ϕ)I(x)]Kσ1∗Hε(ϕ),

(3) f2(x)=Kσ1∗[(1−Hε(ϕ))I(x)]Kσ1∗(1−Hε(ϕ)).

**Figure 2:**
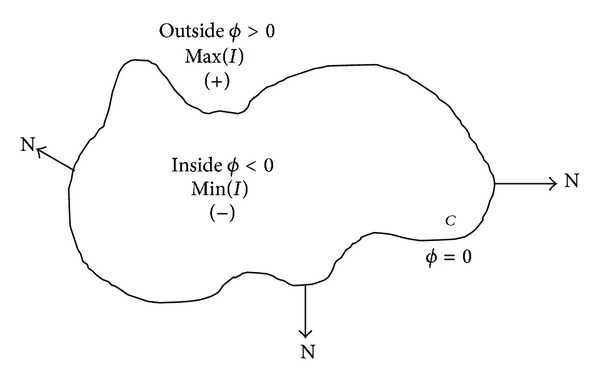
The curve *C* = {*x* : *ϕ*(*x*) = 0} propagating in normal N direction.

In image segmentation, active contours are dynamic curves that move toward the object boundaries to partition an image into nonoverlapping regions. To achieve this goal, we explicitly define an energy functional that can move the zero level curve toward the object boundaries.  Figure 2 illustrates the above assumptions and notations on the level set function *ϕ* defining the evolving curve *C*, where at boundary of curve *C* value of *ϕ* = 0 and our level set function moves inwards or outwards, based on the signs of the SPF for the further evolution. We define energy functional containing an edge-based length term and a region-based area term for function *ϕ* as follows:

(4) $$\begin{array}{c} E_{g,\text{spf}}\left(\phi \right)=\lambda L_{g}\left(\phi \right)+vA_{\text{spf}}\left(\phi \right), \end{array}$$

where *λ* > 0 and *v* are constants and the terms *L*_*g*_(*ϕ*) and *A*_spf_  (*ϕ*) are defined as follows:

(5) Lg(ϕ)=∫Ωg(I)δε(ϕ)|∇ϕ|dx,

(6) Aspf(ϕ)=∫Ωspf(I)Hε(−ϕ)dx,

respectively. Here, *δ*_*ε*_ = *H*_*ε*_′ is the univariate Dirac function and *H*_*ε*_ is the Heaviside function defined in (9) and (10), respectively, while *g*(*I*) is edge indicator function and spf(*I*) is locally computed SPF function defined in (11) and (14), respectively. The zero level curve *C* is driven into a smooth curve from a complicated curve to minimize the function *L*_*g*_(*ϕ*) which utilizes edge information in regularization process, while *A*_spf_(*ϕ*) contains the locally computed image intensity information which derives the contour to the weak and blur edges by distinguishing inhomogeneous regions.

The energy *E*_*g*,spf_(*ϕ*) drives the zero level set toward the object boundaries. The coefficient *v* of *A*_spf_(*ϕ*) in (4) can be positive or negative, depending on the relative position of the initial contour to the object of interest. For example, if the initial contours are placed outside the object, the coefficient *v* in the weighted area term should take a positive value, so that the contour can shrink faster. If the initial contours are placed inside the object, the coefficient *v* should take a negative value to speed up the expansion of the contours. By the calculus of variations [16], the Gateaux derivative (first variation) of the functional *E*_*g*,spf_  (*ϕ*) in (4) can be written as

(7) $$\begin{array}{c} Q\left(\phi \right)=\lambda \delta_{\varepsilon }\left(\phi \right)\mathrm{div}\left(g\left(I\right)\cdot \frac{\nabla \phi }{\left|\nabla \phi \right|}\right)+v\cdot \text{spf}\left(I\right)\delta_{\varepsilon }\left(\phi \right). \end{array}$$

The function *ϕ* that minimizes this functional satisfies the Euler Lagrange equation ∂*E*_*g*,spf_/∂*ϕ* = 0. A classical iterative process for minimizing the function is the gradient flow with artificial time *t* given as

(8) ϕ(t=0)=ϕ0,∂ϕ∂t=Q(t).

After evolving the level set using (7) and (8) we smooth it by using *ϕ* ^*k*^ = *G*_*σ*_2__∗*ϕ* ^*k*^. It not only regularizes the level set function but also eliminates the need of reinitialization, which is computationally very expensive. Moreover, it gives energy leakage free reinitialization.

In the proposed work, the Dirac function *δ*_*ε*_ and Heaviside function *H*_*ε*_ used in (2), (3), and (7) are the smoothed version of the Dirac function and Heaviside function of the entire region. The approximations *δ*_*ε*_ and *H*_*ε*_ as proposed in [6] are

(9) δε(z)=επ(z2+ε2),

(10) Hε(z)=12(1+2πarctan(zε))

and the edge indicator function *g*(*I*) is a positive and strict decreasing function defined follows:

(11) $$\begin{array}{c} g\left(I\right)=\frac{1}{1+{\left|\nabla G_{\sigma_{1}}*I\right|}^{2}}. \end{array}$$

In outmoded level set methods, it is essential to initialize the level set function *ϕ* as a signed distance function (SDF) *ϕ*_0_. If the initial level set function is expressively different from the SDF, then the reinitialization schemes are unable to reinitialize the function to the SDF. In our formulation, not only is the reinitialization procedure completely eliminated but also the level set function *ϕ* no longer needs to be initialized as an SDF. The initial level set function *ϕ*_0_ is defined as

(12) $$\begin{array}{c} \phi \left(x,t=0\right)=\{\begin{array}{cc} -\rho , & x\in \Omega_{0}-\partial \Omega_{0},\\ 0, & x\in \partial \Omega_{0},\\ \rho , & x\in \Omega -\Omega_{0}, \end{array} \end{array}$$

where *ρ* > 0 is a constant and we use *ρ* = 1.

Finally, the principle steps of the algorithm can be summarized as follows.

- Initialize level set function *ϕ* with −*ϕ*_0_ using (12) at *k* = 0.
- Compute edge indicator function *g*(*I*) using (11).
- Compute local mean values *f*_1_ and *f*_2_ from (2) and (3), respectively, where *σ*_1_ is the standard deviation of the truncated Gaussian kernel used to compute *f*_1_ and *f*_2_.
- Calculate SPF function spf(*I*) using (14).
- Solve the partial differential equation (PDE) in *ϕ* from (7) and (8), to obtain *ϕ* ^*k*^.
- Regularize the level set function by a Gaussian kernel; that is, *ϕ* ^*k*^ = *G*_*σ*_2__∗*ϕ* ^*k*^, where *σ*_2_ is standard deviation of a Gaussian kernel.
- Check whether solution is stationary and if not, go to step (ii), *k* = *k* + 1, and repeat.

## 3. Locally Computed SPF Function

The SPF function defined in [17] has values in the range [−1, 1]. It modulates the signs of the pressure force inside and outside the region of interest so that the contour shrinks when outside the object and expands when inside the object. Traditional SPF is formulated using global properties of image; therefore, it works poorly with intensity inhomogeneous images. Here, we introduce a new SPF function based on the local properties of image inside and outside of the contour. This newly formulated SPF function formulates the signs of the signed pressure force function inside and outside the boundary of the region of interest using locally computed mean values. A local fitted image formulation is defined as

(13) $$\begin{array}{c} I_{\text{LFI}}=f_{1}H_{\varepsilon }\left(\phi \right)+f_{2}\left(1-H_{\varepsilon }\left(\phi \right)\right). \end{array}$$

Using the above defined local fitted image we construct the SPF function as follows:

(14) $$\begin{array}{c} \text{spf}\left(I\right)=\{\begin{array}{cc} \frac{I\left(x\right)-I_{\text{LFI}}}{\max\left(\left|I\left(x\right)-I_{\text{LFI}}\right|\right)}, & I\left(x\right)\neq 0,\\ 0, & I\left(x\right)=0. \end{array} \end{array}$$

**Figure 3:**
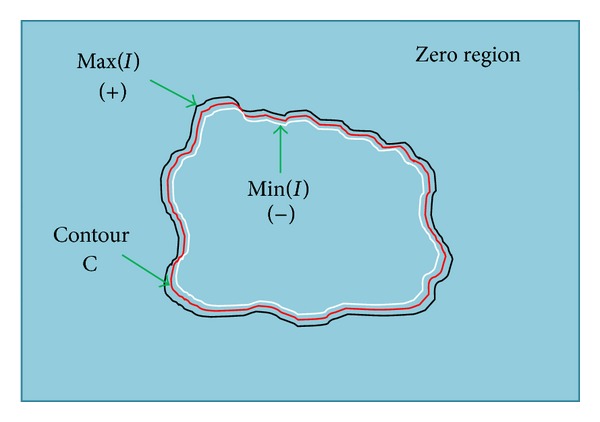
Locally computed SPF function with opposite sign inside and outside the boundary of the region of interest to be segmented and remaining as zero region.

The terms *f*_1_ and *f*_2_ are defined in (2) and (3), respectively. The SPF function computed using the local properties of image is shown in Figure 3, in which black color line shows the positive values of SPF function which are outside the boundary of the region of interest, while white color line shows the negative values of SPF function which are inside the boundary of the region of interest. Red color line shows the position of final level set curve which will be in between the negative and positive values of SPF function and the remaining region of Figure 3 is the zero region with values equal to 0.

The sign and value of SPF function ranges in [−1  1] for both local and global SPF functions; the only difference is the construction method used. As mentioned earlier global SPF function uses global mean values inside and outside the contour; that is why it cannot distinguish between the inhomogeneous changes in the intensity. Therefore, it assigns values with the same sign to both inner and outer region when the intensity change inside and outside region is not distinctive to the global intensity mean computing function. On the other hand local SPF function uses local mean value inside and outside the contour which helps to distinguish between inhomogeneous changes in the intensity. The local intensity mean values are computed by utilizing the Gaussian kernel. It helps computing local maxima for intensity inhomogeneity region which global model fails to find. It offers different signs for both inside and outside the region although there is inhomogeneous intensity change between inside and outside the region.

**Figure 4:**
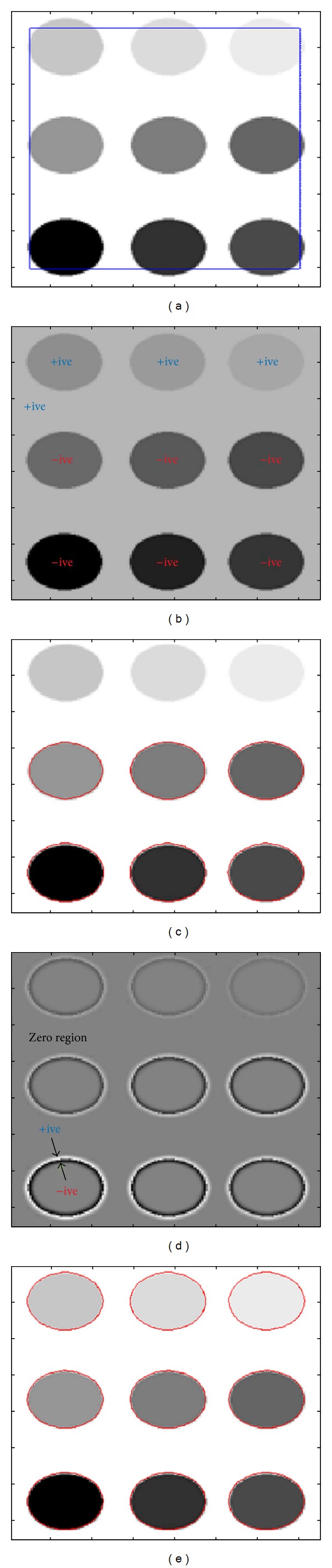
Global and local SPF function sign and result comparison. (a) Initial contour; (b) global SPF function; (c) segmentation result with active contour method using global SPF function; (d) local SPF function; (e) segmentation result with active contour method using local SPF function.

Figure 4 illustrates the signs and segmentation result comparison of local and global SPF function based active contour models.  Figure 4(a) shows the image with initial contour for both local and global cases.  Figure 4(b) shows the signs of global SPF function, in which we can see that for the inhomogeneous intensity change in the image the sign assigned to inside and outside the regions are the same, while for distinctive change in intensity by the global mean computing function sign assignment for both inside and outside the region is different.  Figure 4(c) shows the segmentation result using global SPF based active contour model. Comparing the position of final contour with Figure 4(b) we can see that final contour is positioned where the sign changed from positive to negative between two regions.  Figure 4(d) shows the sign of local SPF function, in which at the boundary inside the region SPF function has value with negative sign while at the boundary outside the region it has positive value. But the positive and negative values are not spread throughout the region inside and outside of the object, instead these are restricted near the boundary. The remaining region is called zero region with zero values of SPF function.  Figure 4(e) shows the segmentation result using local SPF based active contour model. Comparing the position of final contour with Figure 4(d) we can see that final contour is positioned where the sign changed from positive to negative between two regions. From the results we can see that using the active contour model with local SPF function intensity inhomogeneous regions are segmented well while active contour model with global SPF function failed to do so.

## 4. Result Analysis and Comparison

### 4.1. 2D Synthetic and Brain MR Image Segmentation Results

The proposed method is implemented using MATLAB 7.12, in Windows 7 environment on a 2.4 GHz Intel Quad-Core personal computer with 8 GB of RAM. The range of intensities in all images is represented from 0 to 255, while the size in pixels (length × width) of images is variable for all images. In this section we applied the proposed method to synthetic and real images of different modalities and used the parameters which are *λ* = 1, *v* = 22, *ρ* = 1, *ε* = 1.5, *σ*_1_ = 5, *σ*_2_ = 1, *K* = 5, and *τ* = 1, where *v* is force term constant which controls the contour evolution speed and time complexity of desired contour. *σ*_1_ is standard deviation of the truncated Gaussian kernel *K*_*σ*_1__(*x*) with the size 4*k* + 1 by 4*k* + 1. *σ*_2_ is the standard deviation of the smoothing Gaussian kernel which is used to regularize the level set. Selection of *σ*_1_ and *σ*_2_ may be different for different types of images. If we select small values for *σ*_1_ and *σ*_2_ then contour will evolve faster but it will not be accurate; that means small values of *σ*_1_ and *σ*_2_ can reduce the time complexity but can decrease the accuracy. For large values of *σ*_1_ and *σ*_2_ time complexity will increase but segmentation accuracy will also increase. In case of noisy images selection of *σ*_2_ should be bigger than normal to smooth the level set curve.

**Figure 5:**
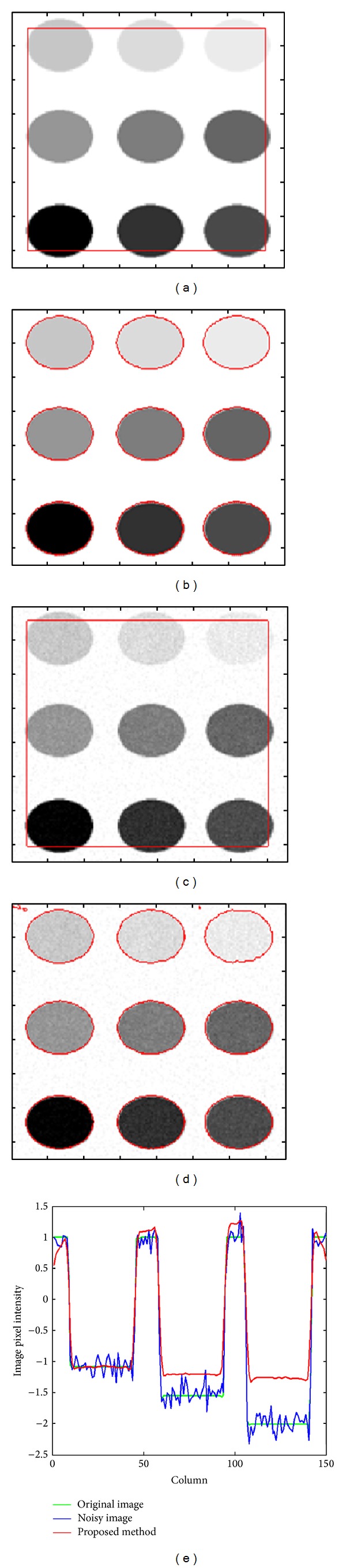
Segmentation results on synthetic images with and without noise. (a) Initial contour for clean image; (b) segmentation result of clean image; (c) initial contour for noisy image; (d) segmentation result of noisy image; (e) profile selection of the middle rows of the original image (the green solid line), noisy image (the blue solid line), and final contour using the proposed method (the red solid line).

Figure 5 shows the segmentation result on a synthetic image with nine different intensities.  Figure 5(a) is the initial contour; Figure 5(b) is the segmentation result of synthetic image without noise. Obviously, these objects with different intensities both homogeneous and inhomogeneous are successfully extracted because the proposed method also works well with the intensity inhomogeneity. We then add Gaussian noise to the clean synthetic image. The noisy image that is shown in Figures 5(c) and 5(d) shows the corresponding segmentation result of our method on the noisy image. From the segmentation results obtained from both clean and noisy synthetic image we can see that segmentation result for both clean and noisy image is similar, which implies that the proposed method segments well under the dense Gaussian noise as applied in this case.  Figure 5(e) displays the central row intensity profile of the input synthetic image with both clean and noisy data along with the final contour. It shows that irrespective of data the resultant contour followed the edges perfectly. In Figure 5(e) we normalized the intensity scale to [−1  1] in order to visualize data from input image profile and final contour profile at the same time with same peak values. The number of iterations used to evolve contour from initial to final form is 200.

**Figure 6:**
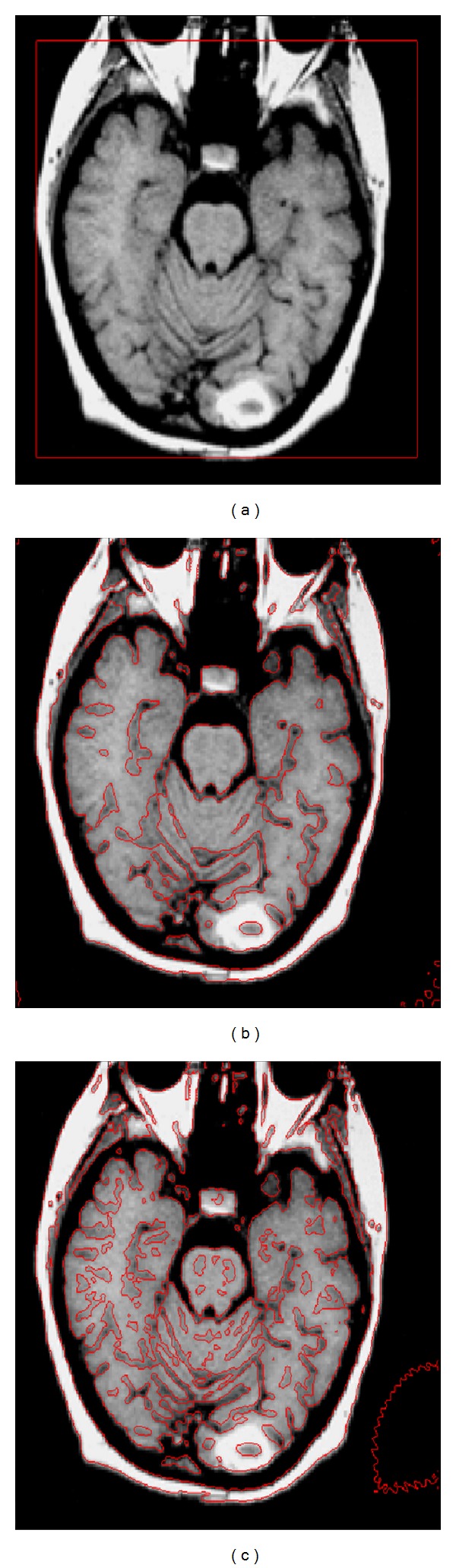
2D brain MR image segmentation using different values of σ_2_. (a) Initial contour; (b) final contour with σ_1_ = 3.0 and σ_2_ = 1.0; (c) final contour with σ_1_ = 3.0 and σ_2_ = 0.5.

Figure 6 shows the importance of *σ*_2_ in the proposed model using a real brain MR image.  Figure 6(a) is the initial contour, Figure 6(b) is the final contour with *σ*_2_ = 1.0, and Figure 6(c) is the final contour with *σ*_2_ = 0.5. From Figures 6(b) and 6(c) we can see that if we choose large value of *σ*_2_ then the proposed method does not segment small details, while the selection of small *σ*_2_ makes segmentation algorithm more sensitive to noise. By using the small value of *σ*_2_ we can segment more detailed objects. For the blurry images *σ*_2_ should be small and for noisy image *σ*_2_ should be large. The total number of iterations used in the contour evolution process is 150.

**Figure 7:**
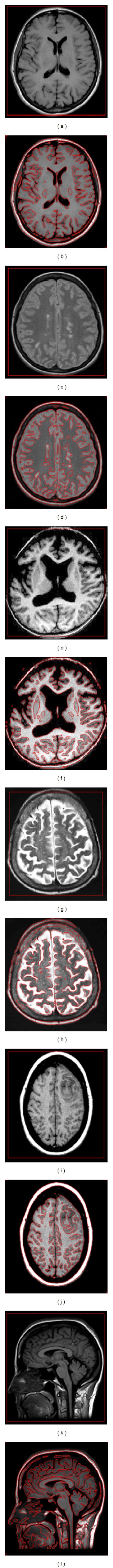
2D brain MR image segmentation results using the proposed method. (a), (c), (e), (g), (i), and (k) initial contour. (b), (d), (f), (h), (j), and (l) final contour.

Segmentation of brain MR image into disjoint regions based on white matter (WM), gray matter (GM), and cerebral spinal fluid (CSF) is a well-known problem. Due to the geometric complexity of the human brain cortex, manual slice by slice segmentation is quite difficult and time consuming [18]. Numerous methods of image segmentation have been developed to solve such problems [19]. Active contour method is one of those methods which are used in this context. Because of the complex intensity inhomogeneous regions, brain MR images are hard to segment successfully with high accuracy [20]. The proposed method is formulated in the context of intensity inhomogeneity problem. To show the robustness and effectiveness of the proposed algorithm for inhomogeneous images we applied it on 2D real brain MR images with intensity inhomogeneity.  Figure 7 shows brain MR image segmentation results using the proposed method. Figures 7(a), 7(c), 7(e), 7(g), 7(i), and 7(k) show the initial contours and Figures 7(b), 7(d), 7(f), 7(h), 7(j), and 7(l) show their respective final contours. The segmentation results from Figure 7 show that the proposed method works very well for the image with the intensity inhomogeneity. The total number of iterations used in contour evolution of Figures 7(b), 7(d), 7(f), 7(h), 7(j), and 7(l) are 400, 400, 450, 250, 400, and 500, respectively.

### 4.2. 3D Brain MR Image Segmentation Results

**Figure 8:**
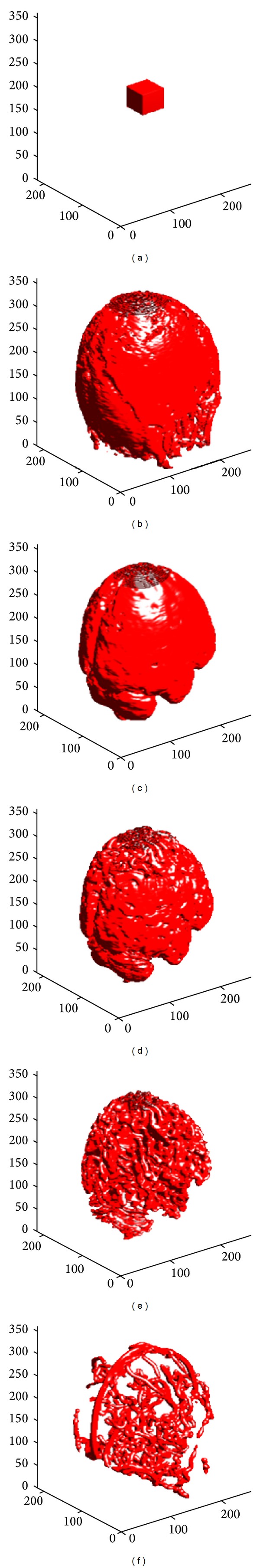
3D brain MR image segmentation results using the proposed method with different anatomical models. (a) Initial contour; (b) skull; (c) CSF; (d) gray matter; (e) white matter; (f) blood vessels of brain.

In this section segmentation results of different brain regions are displayed using 3D brain MR models [21] by applying the proposed method. The range of intensities in all images is represented from 0 to 255, while the size in voxels (length × width × height) of images is (217 × 260 × 362). We have chosen the models of five different regions of head which are involved in 3D brain MR scan of a human test subject [21].  Figure 8 shows the segmentation using five anatomical models of human subject in which Figure 8(a) shows the initial contour, Figure 8(b) shows the final contour of skull model, Figure 8(c) shows the final contour of CSF (cerebral spinal fluid) model, Figure 8(d) shows the final contour of the gray matter region model, and Figure 8(e) shows the final contour of the white matter region model, while Figure 8(f) shows the final contour of the blood vessels in head. We can see some circular artifacts in Figures 8(b)–8(e), which are here because of the noise present at the time of image acquisition. We can remove this artifact by applying smoothing kernel on the data input before using the segmentation algorithm. We have applied the proposed method on 3D MR Dataset to show its application in volume visualization and data exploration.

### 4.3. Comparison with Traditional Active Contour Methods Using SPF Function in Their Model

Zhang et al. in [7] and Jiang et al. in [8] used SPF function in their proposed method but their methods cannot segment well the images with intensity inhomogeneity. The SPF function they used in their model is grounded on CV region-based active contour method which computes mean of intensity globally that is not sufficient in order to segment the images with intensity inhomogeneity, while the proposed SPF function uses local mean value which provides well segmentation results for images with intensity inhomogeneity but with drawback of high time complexity [22]. In order to show advantages of the proposed method over other active contour methods which utilize traditional global SPF function in their models, we compare their results using a synthetic image with intensity inhomogeneity. The parameters used for this comparison for Zhang et al. method are *μ* = −25, *ρ* = 1, *ε* = 1.5, *σ* = 1, *K* = 5, and Δ*t* = 1, while the parameters used for Jiang et al. method are *μ* = 0.04, *λ* = 3, *v* = 1, *ρ* = 2, *ε* = 1.5, and *τ* = 5  and  the parameters used for the proposed method are same as described in Section 4.1 with *σ*_1_ = 1.

**Figure 9:**
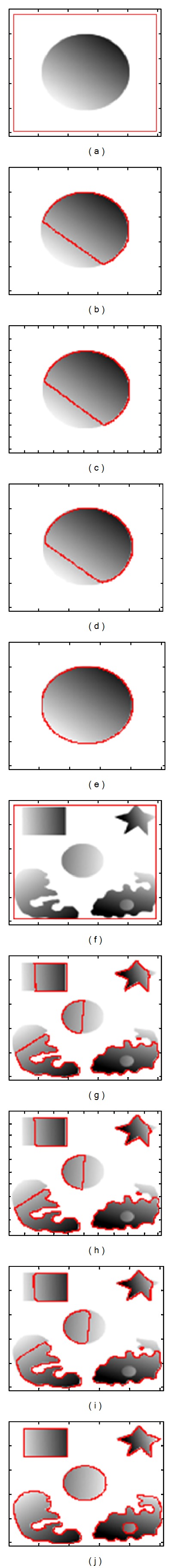
Comparison of segmentation results using synthetic image with intensity inhomogeneity with other active contour methods that use traditional SPF function. (a) and (f) initial contour; (b) and (g) CV energy model; (c) and (h) Zhang et al. method; (d) and (i) Jiang et al. method; (e) and (j) the proposed method.

Figure 9 shows a segmentation result comparison with other active contour methods which use traditional global SPF function in their model. In that figure we can see that the methods using traditional global SPF function cannot segment well when image has intensity inhomogeneous region in it. In Figures 9(b) and 9(g) CV energy model could not properly segment all objects with intensity inhomogeneity. Figures 9(c) and 9(h) show that Zhang et al. method that uses SPF function in its model, which is constructed using global mean values from the CV energy model, also could not correctly segment inhomogeneous regions. Figures 9(d) and 9(i) show that Jiang et al. method using same SPF function used by Zhang et al. also could segment well all objects with intensity inhomogeneity, while the proposed method that uses new SPF function, which uses means from local neighborhood, could accurately segment all inhomogeneous objects in both Figures 9(e) and 9(j).

The proposed method can segment image with intensity inhomogeneous regions yet it takes additional processing time as compared to the active contour methods using traditional CV based SPF function.  Table 1 provides the processing time comparison of the proposed method with the Chan et al., Jiang et al., and Zhang et al. methods which show that although the proposed method segments all regions well, its time complexity is higher than the other methods which use CV region-based global SPF function in their model.

### 4.4. Comparison with the LBF, LIF, and CV Energy Models

In this paper we proposed a region-based active contour method using locally computed SPF function. The proposed method works similar to previously developed LBF [11, 12] and LIF [13] energy models. To show the effectiveness, accuracy, and robustness of the proposed algorithm we compared the segmentation results with the LBF, LIF, and CV energy models. For the comparison we used both synthetic and real brain MR images. The parameters used for this comparison for the LBF energy model are *λ*_1_ = 1, *λ*_2_ = 1, *μ* = 1, *v* = 0.001 × 255^2^, *ρ* = 1, *ε* = 1.5, *σ* = 4, *K* = 5, and *τ* = 0.1. The parameters used for the LIF energy model are *σ*_1_ = 5, *σ*_2_ = 1, *K* = 5, *ρ* = 1, *ε* = 1.5, and *τ* = 1. The parameters used for the CV model are *λ*_1_ = 1, *λ*_2_ = 1, *μ* = 0.2, *v* = 1, *ρ* = 1, *ε* = 1.5, and *τ* = 1, while the parameters used for the proposed method are same as mentioned in Section 4.1 with *σ*_1_ = 3.

**Figure 10:**
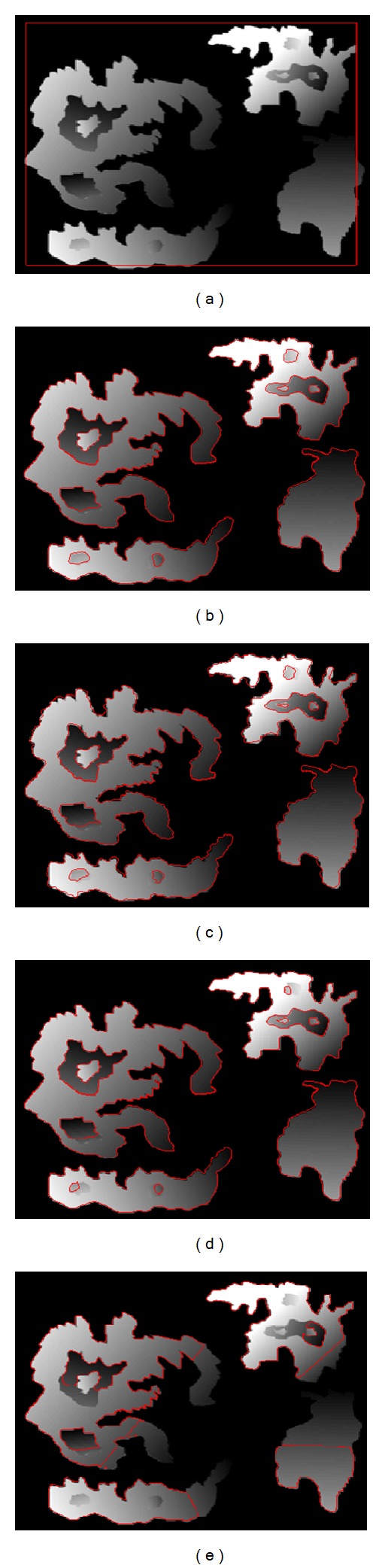
Comparison of segmentation results using synthetic image with intensity inhomogeneity with the LBF, LIF, and CV energy models. (a) Initial contour; (b) the proposed method; (c) LIF energy model; (d) LBF energy model; (e) CV energy model.

Figure 10 shows a comparison between the proposed method, LBF, LIF, and CV energy models using synthetic image.  Figure 10(c) shows that the final contour computed by the LIF energy model could not strictly follow the object boundaries.  Figure 10(d) shows that the LBF energy model could not accurately segment the small inhomogeneous objects.  Figure 10(e) shows that the CV model cannot properly segment intensity inhomogeneous regions; moreover, small objects are also missed in the segmentation process.  Figure 10(b) shows that the proposed method provided better segmentation from the entire state-of-the-art active contour methods used in the comparison.

**Figure 11:**
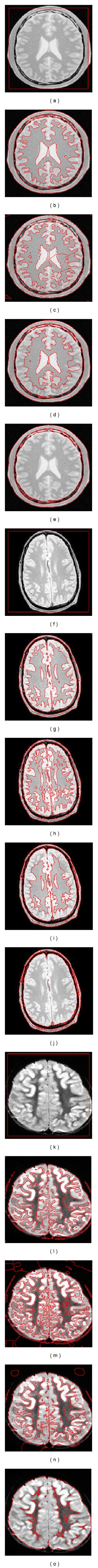
Comparison of segmentation results using real brain MR images with intensity inhomogeneity with the LBF, LIF, and CV energy models. (a), (f), and (k) initial contour; (b), (g), and (l) the proposed method; (c), (h), and (m) LIF energy model; (d), (i), and (n) LBF energy model; (e), (j), and (o) CV energy model.

Figure 11 shows a comparison between the proposed method, LBF, LIF, and CV energy models using three brain MR images. For the comparison shown in Figure 11, parameters for the proposed method are same as mentioned in Section 4.1. The parameters of CV energy model are same as described earlier in this section, while for the LBF and LIF energy models *σ* = 5 and *σ*_1_ = 5, respectively, and the rest of the parameters are same as mentioned earlier in this section.

Figures 11(b), 11(g), and 11(l) show the segmentation results using the proposed algorithm, which, compared to the generated results by other methods, are better in every aspect. There is no over lapping in the region during the segmentation process and contour accurately evolved to the boundary of the object need to be segmented. The proposed method even segmented the sharp details of the objects at the boundary. Figures 11(c), 11(h), and 11(m) display the segmentation results of the LIF energy model. Although the LIF energy model segmented sharp details there are overlapping of contours in the regions during the segmentation process. Figures 11(d), 11(i), and 11(n) show the segmentation results of the LBF energy model. In Figures 11(d) and 11(i) the LBF model segmented well without any overlapping of contours in the regions but it failed to segment the sharp details of the boundary of the region, while in Figure 11(n) there are also some signs of contour overlapping during segmentation process. Figures 11(e), 11(j), and 11(o) display the segmentation result using CV energy model. It shows that the CV energy model is unable to segment the intensity inhomogeneous regions in the brain MR images.

Table 2 shows the time complexity analysis of the proposed method, LBF, LIF and, CV energy models from Figures 10 and 11. In Figures 10 and 11, the proposed method has less time complexity compared to LBF and LIF energy models. For Figure 10, at *σ*_1_ = *σ* = 4.0 the time complexity of LIF method to complete 400 iterations is 22.06 sec which is faster than any other method, whereas the proposed method reached the final contour in 400 iterations in 26.29 sec and the LBF method took 28.87 sec to complete 400 iterations. Although at *σ*_1_ = *σ* = 4.0 LIF method has less time complexity as compared to the state-of-the-art methods, there is overlapping over regions in segmented image of LIF method. It shows the importance of *σ*_1_ selection and how it can affect the time complexity of the method. However, for Figures 10 and 11(k) the CV energy model has the less time complexity than any other method, as CV energy model cannot properly segment images with intensity inhomogeneity; the proposed method being second in time complexity is still the best among remaining methods. *σ*_1_ plays an important role in time complexity and segmentation accuracy. If we select same value of *σ*_1_ = *σ* the proposed method has less time complexity and better segmentation results compared to the state-of the-art locally computed active contour methods.

### 4.5. Quantitative Analysis

**Figure 12:**
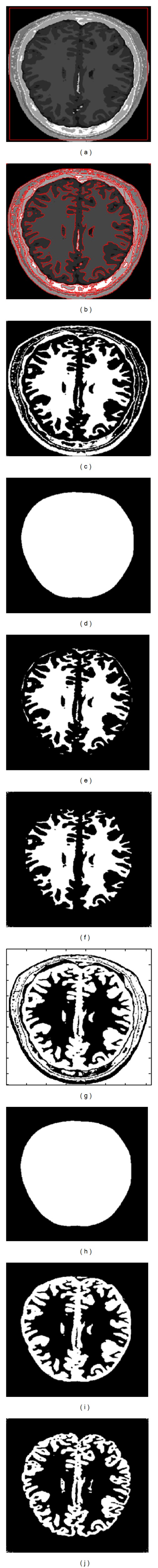
Visual based segmentation accuracy analysis of WM and GM regions. (a) Initial contour; (b) final contour; (c) WM region of final contour which is phase at *ϕ* > 0; (d) hand drawn brain mask; (e) WM region after brain mask scaling; (f) WM region ground truth; (g) GM region of final contour which is phase at *ϕ* < 0; (h) hand drawn brain mask; (i) GM region after brain mask scaling; (j) GM region ground truth.

As discussed earlier white matter (WM) and gray matter (GM) are the main regions of interest in the segmentation of brain MR images. In order to segment WM and GM we split the segmentation result into two regions based on two phases. WM region represents the phase at *ϕ* > 0 and the GM represents the phase at *ϕ* < 0. The WM and GM regions represent the brain region which is the region of interest, while the regions outside the brain, for example, skull, fats, and vacuum, can be taken as unnecessary regions. Therefore, we have used a hand drawn brain mask to extract the WM and GM only and remove the other needless regions outside the brain.  Figure 12 shows the computed WM and GM from the proposed method phases and compares it with the given ground truth. It shows how we extracted only GM and WM regions by scaling it to the brain area using hand drawn brain mask.  Figure 12(a) shows the initial contour at *t* = 0. After some time *t* = *n*, we obtained the final contour shown in Figure 12(b). Then we divided the final contour into two phases based on the value of *ϕ*; at *ϕ* > 0 we acquired WM region and at *ϕ* < 0 we obtained GM region as shown in Figures 12(c) and 12(g), respectively. Figures 12(d) and 12(f) show hand drawn brain mask. After obtaining WM and GM regions from two phases of final contour we then multiply these regions with hand drawn brain mask in order to scale them to brain area only and remove other regions outside the brain, for example, skull, fats, and vacuum, as shown in Figures 12(e) and 12(i), respectively. Finally, we compare the scaled WM and GM regions computed using the proposed method with their respective ground truths to visually analyze the segmentation accuracy of the proposed method.

In order to do the quantitative analyses we used the ground truths of 2D slices from the 3D anatomical brain models [21]. The quantitative analysis for the proposed method, LBF, and LIF models is shown in Table 3 using the following expression for the percentage accuracy:

(15) $$\begin{array}{c} \text{percentage}\;\text{accuracy}=\frac{A\cap B}{A\cup B}\%. \end{array}$$

We compute the accuracy by using the given ground truth data and the computed segmentation results. In the above expression *A* is the ground truth of the WM or GM region and *B* is the brain mask scaled WM or GM region from *ϕ* > 0 or *ϕ* < 0, respectively, using the proposed method, LBF model, and LIF model. Table 3 shows that the proposed method provides better segmentation accuracy as compared to other locally computed active contour methods. It provides average segmentation accuracy of 91.04% and 74.28% in case of WM and GM regions, respectively, while LBF model provides average segmentation accuracy of 76.74% and 64.96% in case of GM and WM regions, respectively, and LIF model provides average segmentation accuracy of 60.50% and 51.06% in case of WM and GM regions, respectively. This shows that the proposed method has high percentage accuracy for both WM and GM regions as compared to LBF and LIF models.

Selection of standard deviation *σ* of truncated Gaussian kernel plays an important role both in the time complexity and segmentation accuracy of the algorithm. If *σ* or *σ*_1_ is big then contour evolves faster (less number of iterations) to its final form with much of segmentation accuracy but its time complexity also increases. If *σ* or *σ*_1_ is small then contour evolves slower (more number of iterations) to its final form but there can be segmentation accuracy problem. *σ*_2_ is used to regularize the contour and remove the need of initialization. The bigger *σ*_2_ is the smoother the contour would be at the boundary of the object to be segmented.

## 5. Conclusion

In this paper a region-based active contour method is presented which embeds both edge-based and region-based terms in its model. As the proposed model contains both edge-based and regions-based terms, it works better than traditional region-based methods and segments well images with weak and blur edges. A new SPF function is formulated which utilizes image local information and helps to segment intensity inhomogeneous regions. The proposed method is applied to the 2D synthetic and real brain MR images with intensity inhomogeneity to show its robustness and effectiveness. We also applied the proposed method to 3D brain anatomical models to show its application in volume visualization and data exploration.

A comparison is shown with other active contour methods that use traditional global SPF function formulated by CV model, using synthetic image with intensity inhomogeneity. It shows that the proposed method accurately segments images with intensity inhomogeneity unlike previously formulated SPF based active contour methods. However, it has higher time complexity than the active contour methods using traditional global SPF function.

We also compared the proposed method with the previously formulated locally computed active contour methods to show the advantages of the proposed method. The visual comparison shows that the proposed method generates better segmentation results as compared to the state-of-the-art active contour methods for both synthetic and real brain MR images. Moreover, it also has less time complexity compared to the local based active contour methods.

Global region based active contour method is fast as compared to the local active contour method but it does not work well for the images with intensity inhomogeneity. On the other hand local active contour method using local maxima can segment images with intensity inhomogeneity but it has high time complexity and it is sensitive to noise. In future we will address the segmentation problem with an active contour method by using a new SPF function that uses both local and global information. By using that we want to target advantages of both local and global region based models.

## Figures and Tables

**Table 1:** Time complexity analysis of methods compared in Figure 9 in terms of CPU time/s.

Figures	Chan et al. method	Zhang et al. method	Jiang et al. method	Proposed method	Number of iterations
Figure 9(a)	8.39	21.22	11.45	13.45	500
Figure 9(f)	6.84	12.8	7.52	8.92	300

**Table 2:** Time complexity analysis of methods compared in Figures 10 and 11 in terms of CPU time/s.

Figures	Proposed method	Zhang et al. method	Li et al. method	Chan et al. method	Number of iterations
Figure 10	24.25	41.92	32.34	11.81	500
Figure 11(a)	7.56	13.66	16.48	18.73	150
Figure 11(f)	25.77	43.52	51.69	34.94	400
Figure 11(k)	82.44	117.53	151.39	65.34	600

**Table 3:** Quantitative analysis to evaluate the segmentation accuracy of GM and WM regions.

Test data	Proposed method		LBF		LIF		Slice number
	Accuracy WM %	Accuracy GM %	Accuracy WM %	Accuracy GM %	Accuracy WM %	Accuracy GM %	
4	85.71	72.74	70.07	64.15	54.65	51.81	144
	94.58	77.97	87.09	73.35	69.55	55.06	200
5	85.48	69.65	76.47	65.21	67.89	59.95	150
	93.58	73.93	75.18	61.27	50.04	37.78	200
	78.44	66.41	70.91	63.15	33.52	40.62	250
6	94.64	84.75	81.82	73.69	82.43	74.64	200
18	95.95	81.90	79.42	70.67	70.46	60.38	200
20	94.75	72.47	76.96	62.07	68.49	53.26	200
38	88.95	69.84	77.64	64.78	63.96	54.31	200
47	92.17	73.29	77.85	64.41	59.55	46.18	200
50	93.45	75.53	60.25	48.47	51.66	38.44	200
54	94.73	72.89	87.19	68.28	53.75	40.32	200
